# SV2A PET Imaging Detects Severity-Dependent Synaptic Changes After Experimental Traumatic Spinal Cord Injury

**DOI:** 10.2967/jnumed.125.271236

**Published:** 2026-05

**Authors:** Claudia Schrauwen, Nicolas Halloin, Annemie Van Eetveldt, Zoë Laermans, Winnok H. De Vos, Aleksandar Jankovski, Marleen Verhoye, Steven Staelens, Charles Nicaise, Daniele Bertoglio

**Affiliations:** 1 Bio-Imaging Lab, University of Antwerp, Antwerp, Belgium;; 2 µNeuro Center for Excellence, University of Antwerp, Antwerp, Belgium;; 3 URPhyM, NARILIS, Université de Namur, Namur, Belgium;; 4 Molecular Imaging Center Antwerp, University of Antwerp, Antwerp, Belgium;; 5 Laboratory of Cell Biology and Histology, University of Antwerp, Antwerp, Belgium;; 6 Antwerp Centre for Advanced Microscopy, University of Antwerp, Antwerp, Belgium; and; 7 Department of Neurosurgery, Vivalia Hospital, Arlon, Belgium

**Keywords:** synaptic vesicle glycoprotein 2A, ^18^F-SynVesT-1, contusion SCI, structural MRI, autoradiography

## Abstract

Traumatic spinal cord injury (SCI) is a serious neurologic disorder that can result in long-term motor and sensory impairments after external damage to the spinal cord. Although anatomic MRI offers essential structural information, it falls short in capturing functional alterations, particularly synaptic changes, which are crucial for accurate prognosis and evaluating therapeutic outcomes. Here, we used ^18^F-SynVesT-1 PET imaging to measure noninvasively synaptic vesicle glycoprotein 2A (SV2A) loss in a rat model of SCI to determine whether SV2A PET is capable of detecting SCI severity-dependent synaptic loss. **Methods:** Rats were subjected to laminectomy (sham, *n* = 10) or unilateral cervical (C5) contusion injury with graded severity (100 kDyn, *n* = 10; 250 kDyn, *n* = 10; and 400 kDyn, *n* = 10). Longitudinal ^18^F-SynVesT-1 PET/CT imaging and structural MRI were performed at 1 and 6 wk after SCI. SV2A immunostaining and ^3^H-SynVesT-1 autoradiography were conducted to validate in vivo PET findings. **Results:** Structural MRI revealed lesion volumes proportional to contusion severity in SCI rats at both 1 wk (*P* < 0.0001) and 6 wk (*P* = 0.0190) after SCI, with overall lesion volume decreasing over time. ^18^F-SynVesT-1 PET imaging detected a significant reduction in SV2A at the injury epicenter proportional to injury severity: −37.5 ± 5.5% (100 kDyn), −39.2 ± 3.7% (250 kDyn), and −41.9 ± 3.5% (400 kDyn) at 1 wk after SCI compared with sham controls (*P* < 0.0001). At 6 wk after SCI, the SV2A loss remained sustained, showing no progression over time. Postmortem immunofluorescence and autoradiography confirmed SV2A loss to be proportional to injury severity and strongly correlated with the in vivo PET. **Conclusion:** ^18^F-SynVesT-1 PET imaging provides a sensitive, noninvasive measure of SV2A loss after SCI and can discriminate between different levels of injury severity. Together, these findings support SV2A PET as an early objective biomarker for SCI severity and progression, offering a valuable tool for evaluating new therapies and further encouraging clinical application of SV2A PET for SCI assessment.

## Visual Abstract

**Figure:**
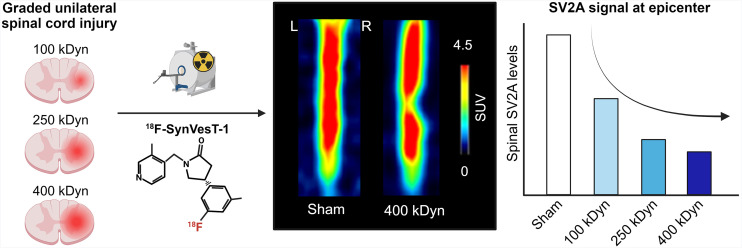

Spinal cord injury (SCI) is a debilitating neurologic condition that disrupts neural circuits, leading to loss of motor, sensory, and autonomic functions, which profoundly affect quality of life and impose a significant societal burden (1). In traumatic SCI, external physical forces damage the spinal cord, triggering not only the initial mechanical insult but also a cascade of secondary pathophysiologic processes. These include ischemia, neuroinflammation, demyelination, axonal degeneration, and synaptic loss, factors that exacerbate the initial neuronal damages and constrain functional recovery (1,2).

Currently, CT and structural MRI (sMRI) remain the most established tools for evaluating the extent of tissue damages, offering detailed visualization of major anatomic damage such as hemorrhage, edema, and lesion size (3,4). Although they are essential for diagnosis and monitoring macroscopic tissue lesion evolution, their ability to capture pathophysiologic information remains limited (5).

Synapses are essential for maintaining neuronal connectivity and circuit integrity, and their disruption after SCI is not confined to the lesion epicenter but can extend both rostrally and caudally, affecting local spinal circuits and distant supraspinal regions (6–8). Mechanistic drivers such as glutamate-driven excitotoxicity, excessive microglial-mediated synaptic pruning, and Wallerian degeneration contribute to synaptic degeneration, impairing plasticity needed for recovery (9–11). Detecting and quantifying synaptic alterations in vivo is therefore crucial to understanding SCI progression and identifying therapeutic windows.

Synaptic vesicle glycoprotein 2A (SV2A), a protein present in the presynaptic vesicles and involved in neurotransmitter release (12), represents a promising noninvasive PET imaging target to estimate synaptic density in the entire central nervous system both preclinically and clinically (13–15). In the SCI context, we previously reported SV2A PET imaging using the radioligand ^11^C-UCB-J as a noninvasive marker for the detection of unilateral cervical spinal damage in experimental models of SCI (16). Recently, those findings were corroborated by another study reporting SV2A PET changes in a thoracic model of SCI (17). However, given the large heterogeneity in the injury severity observed in patients with SCI (2), it remains unknown whether SV2A PET imaging can distinguish between graded severities of SCI. This knowledge is critical to understand the role SV2A PET might have in tracking disease progression and assessing therapeutic interventions across a broader injury spectrum.

Among the available SV2A radioligands, we selected the fluorinated ^18^F-SynVesT-1 given its optimal pharmacokinetics and imaging reliability in both rodents (14,15) as well as humans (18,19), underscoring its translational potential. Specifically, we performed longitudinal SV2A PET at 1 and 6 wk after SCI in rats after unilateral cervical (C5) contusion injury with graded severity to evaluate whether ^18^F-SynVesT-1 PET imaging could distinguish severity-dependent synaptic changes and whether SV2A levels would change over time. Additionally, sMRI was performed to quantify lesion volume and assess tissue integrity, whereas postmortem SV2A immunofluorescence and ^3^H-SynVesT-1 autoradiography provided histologic and molecular validation of the in vivo PET findings.

## MATERIALS AND METHODS

### Animals and Experimental Design

All experiments complied with the European Communities Council Directive (2010/63/EU, 86/609/EEC, and 87-848/EEC), were authorized by the local animal ethic committees (University of Namur [UN22-382] and University of Antwerp [ECD2022-37]), and followed the Animal Research: Reporting of In Vivo Experiments guidelines. Nine-week-old female Sprague–Dawley rats (*n* = 40, 205–350 g; Charles River Laboratories) were housed under a 12-h light/dark cycle in a temperature- and humidity-controlled environment with ad libitum access to food and water. Animals were divided equally into 4 groups: (i) sham controls with laminectomy only; unilateral contusion SCI at the right C5 level with a (ii) low (100 kDyn) impact, (iii) medium (250 kDyn) impact, or (iv) high (400 kDyn) contusion-type injury. The experimental design is outlined in Supplemental Figure 1 (supplemental materials are available at http://jnm.snmjournals.org). At 1 (subacute phase) and 6 wk (chronic phase) after SCI, sMRI and SV2A PET scans were acquired. All animals were euthanized at 6 wk after SCI for histologic and autoradiographic assessments.

### Contusion SCI

The unilateral cervical C5 contusion model was induced as previously described (16,20) using graded injury severity (100, 250, or 400 kDyn). The resulting impact force was 103.9 ± 3.3 kDyn (mean ± SD) for the low-impact group, 276.9 ± 12.0 kDyn for the medium-impact group, and 408.5 ± 1.4 kDyn for the high-impact group. A detailed description of the procedure is provided in Supplemental Data.

### Grip Strength Test

Forelimb grip strength was assessed using a rodent grip strength meter (BIOSEB; In Vivo Research Instruments). Animals grasped a horizontal bar with each forelimb separately and were gently pulled until release. The maximal force from 3 trials was averaged for analysis. Measurements were obtained before surgery (baseline) and at 3 d, 1 wk, 2 wk, 4 wk, and 6 wk after SCI.

### sMRI

3-Dimensional anatomic images of the cervical spinal cord were acquired on a 7-T preclinical MRI scanner (Bruker MRI), using a volume resonator coil for radiofrequency excitation and a 2 × 2 channel head receiver coil for signal detection. A detailed description of the procedure is provided in Supplemental Data.

### ^18^F-SynVesT-1 Radiosynthesis

The radiosynthesis of ^18^F-SynVesT-1 was performed as we previously described (14). The radiosynthesis of ^18^F-SynVesT-1 achieved a product purity of greater than 99%, with a molar activity of 216.0 ± 6.1 GBq/µmol (*n* = 8) at the end of synthesis. A detailed description of the procedure is provided in Supplemental Data.

### microPET Imaging

Dynamic microPET/CT imaging was conducted using Siemens Inveon PET/CT scanners (Siemens Preclinical Solution). Animal preparation followed established protocols (16) using isoflurane (IsoFlo; Zoetis; 5% induction, 1.5% maintenance) as sedation. At the start of the 50-min dynamic PET scan, a bolus injection of 29.7 ± 6.7 MBq at an injected mass of 0.59 ± 0.36 µg/kg was administered via an automated pump (Pump 11 Elite; Harvard Apparatus) over a 10-s interval (1 mL/min) in the lateral vein. This radioactivity dose was selected to ensure high-quality imaging with an optimal signal-to-noise ratio while keeping the injected mass below 1.5–2 µg/kg. After the microPET scan, a 10-min CT (80 kV/500 μA) was performed for attenuation correction and anatomic registration. One 250-kDyn animal was excluded at week 6 because of failed acquisition, resulting in 8 animals. Animal numbers and dosing parameters are available in Supplemental Table 1.

### Image Processing and Analysis

PET data were acquired in list-mode format and reconstructed into 31 frames of increasing duration (12 × 10 s, 3 × 20 s, 3 × 30 s, 3 × 60 s, 3 × 150 s, and 7 × 300 s). Images were reconstructed on a [128 × 128 × 159] grid with voxel dimensions of 0.776 × 0.776 × 0.796 mm^3^ using list-mode iterative reconstruction with proprietary spatially variant resolution modeling, using 8 iterations and 16 subsets of the 3D ordered-subset expectation maximization (OSEM 3D) algorithm (21). PET/CT data were processed and analyzed using PMOD software (version 3.6; PMOD Technologies) based on the previously described procedure (16). Volumes of interest in the cervical spinal cord were manually delineated from C3 to C7 on coregistered PET/CT images, with C5 further subdivided into left and right segments, where the right C5 represented the injury epicenter. An additional volume of interest of the lesion area was created on the basis of the subjects’ MR images to capture the actual lesion area (Supplemental Fig. 2). Radioligand uptake was analyzed within the 25–45-min postinjection time window and quantified as SUVs as we previously performed (16). Since our previous SCI PET study showed no SV2A changes at C3 level after severe unilateral C5 contusion (16), C3 was used as reference area and spinal uptake was normalized to C3.

### SV2A Histologic Analysis

SV2A immunostaining was performed as previously described (14). A detailed description of the procedure is provided in Supplemental Data. Histologic images were analyzed using QuPath (version 0.5.1). The SV2A-positive area was quantified within manually delineated gray matter regions of interest. A single, fixed-intensity threshold was applied to calculate the ipsilateral/contralateral ratio as the proportion of thresholded SV2A-positive area in the ipsilateral hemicord relative to the contralateral hemicord. In the figures, data are presented as the relative decrease in SV2A-positive area compared with the contralateral side. For statistical analysis, data from the 250- and 400-kDyn groups were combined because of reduced sample numbers from sections not matching the intended C5 spinal level.

### SV2A Autoradiography

SV2A autoradiography was performed using ^3^H-SynVesT-1 (Novandi Chemistry AB) as previously reported (22). ^3^H-SynVesT-1 molar radioactivity was 962 MBq/µmol, and radiochemical purity was greater than 99%.

Quantification of the gray matter was conducted using Fiji (ImageJ, version 2.1.0). Specific binding of ^3^H-SynVesT-1 was quantified by converting mean gray values to radioactivity density, calibrated using commercial tritium standards (American Radiolabeled Chemicals). Radioactivity density was then converted to binding density for each region based on the molar activity of ^3^H-SynVesT-1 on the day of the experiment.

### Statistical Analysis

Regional analysis to test for group differences for the different metrics at each spinal segment and each time point was assessed using 1-way ANOVA with the false discovery rate method (Benjamini–Hochberg procedure) for multiple comparison correction. Each ANOVA included 4 experimental groups (sham, 100, 250, and 400 kDyn), corresponding to 6 pairwise comparisons per analysis. Correlations between in vivo and postmortem SV2A signals were evaluated using the Spearman (*r*) rank correlation test. All statistical analyses were conducted with GraphPad Prism (version 9.1). Data are presented as mean ± SD unless otherwise stated. All tests were 2-tailed, with statistical significance set at a *P* value of less than 0.05.

## RESULTS

### Spinal Lesion Volume Is Proportional to Contusion Severity

**FIGURE 1.**
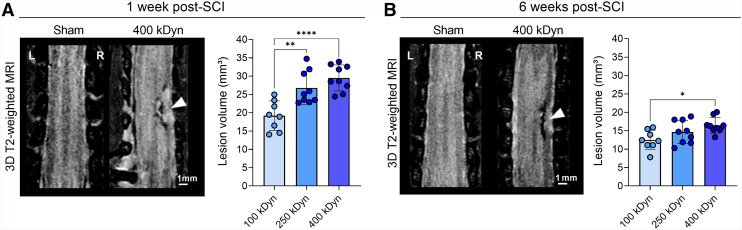
3D T2-weighted MRI reveals graded lesion volumes in rat contusion SCI. (A) Representative axial images from sham and 400-kDyn rat at 1 wk after SCI, with lesion site indicated (arrowhead), alongside corresponding lesion volume bar plots. (B) MR images and lesion volume plots from same animals at 6 wk after SCI. *P < 0.05, **P < 0.01, ****P < 0.0001.

To confirm the correct targeting of the right C5 segment and assess lesion volume across SCI severities, the size of the lesion based on sMRI was quantified at 1 and 6 wk after contusion in rats subjected to graded SCI. During the subacute phase, at 1 wk after SCI, a significant injury severity–dependent lesion volume was observed (group effect: *F*_(2,23)_ = 14.66, *P* < 0.0001; Fig. 1A) with both 250 and 400 kDyn having a significantly different lesion volume compared with the 100-kDyn group (post hoc *P* = 0.0022 and *P* < 0.0001, respectively). The 3D MRI of the sham group did not show any detectable lesion. During the chronic phase, at week 6 after SCI, a statically significant group effect remained (*F*_(2,23)_ = 4.74, *P* = 0.0190; Fig. 1B); however, the post hoc analysis revealed a significantly different lesion volume only between the 100- and 400-kDyn groups (*P* = 0.014).

### SV2A Decrease Extends Beyond Epicenter to Distal Segments of the Cervical Spine

To determine the spatial extent of synaptic alterations after graded contusion SCI, we evaluated SV2A levels in anatomically defined spinal segments using ^18^F-SynVesT-1 PET.

**FIGURE 2.**
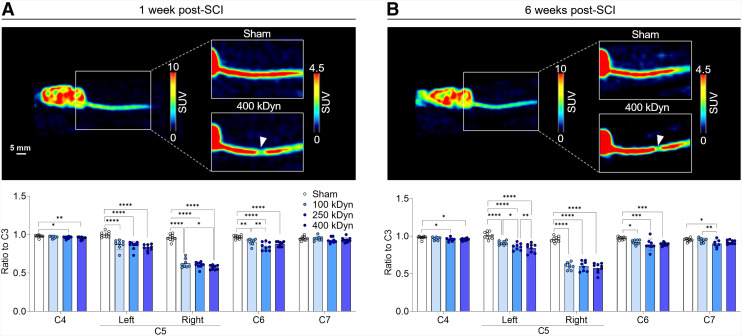
^18^F-SynVesT-1 PET detects graded loss of synaptic density (SV2A) after SCI. (A) Representative uncropped and sagittal PET images at 1 wk after SCI from sham and 400-kDyn rat. Bar plots show ^18^F-SynVesT-1 uptake across cervical segments C4–C7, normalized to C3 segment within each animal. (B) Sagittal PET images of same animals at 6 wk after SCI with corresponding bar plots. Arrowheads indicate C5 level. Interval is 25–45 min after injection. *P < 0.05, **P < 0.01, ***P < 0.001, ****P < 0.0001.

At 1 wk after SCI, significant SV2A reductions were detected ipsilateral at C5 (group effect: *F*_(3,31)_ = 178.5, *P* < 0.0001). Interestingly, SV2A loss was not confined to the C5 epicenter, but it extended to distant spinal regions, with significant changes observed as group effects rostrally at C4 (*F*_(3,32)_ = 4.00, *P* = 0.0159) and caudally at C6 (*F*_(3,32)_ = 17.1, *P* = 0.0003), suggesting subacute involvement of secondary injury mechanisms (Fig. 2A).

Similarly, at 6 wk after SCI, SV2A loss at C5 remained pronounced (group effect: *F*_(3,31)_ = 109.0, *P* < 0.0001), and reductions persisted for both rostral (C4: *F*_(3,31)_ = 3.5, *P* = 0.0271) and caudal segments (C6: *F*_(3,31)_ = 8.5, *P* = 0.0003). Although synaptic loss was most pronounced at the lesion epicenter, a significant reduction also emerged at the C7 segment by 6 wk after SCI (*F*_(3,31)_ = 4.4, *P* = 0.0107), highlighting the sensitivity of ^18^F-SynVesT-1 PET to both primary and delayed secondary synaptic degeneration (Fig. 2B).

### ^18^F-SynVesT-1 PET Detects Severity-Dependent Persistent SV2A Loss

**FIGURE 3.**
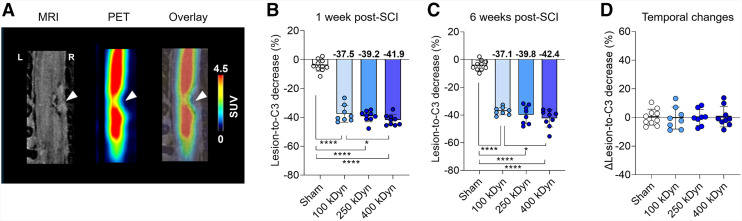
SV2A PET imaging demonstrates severity-dependent persistent loss of synaptic density after injury. (A) At 1 wk after SCI, images from single 400-kDyn animal illustrate lesion epicenter: axial 3D T2-weighted MRI identifies the lesion (left); ^18^F-SynVesT-1 PET reveals synaptic density (middle), and their overlay highlights colocalized synaptic loss (right). (B and C) Bar plots show normalized tracer uptake at C5 (lesion) relative to C3 at both 1- and 6-wk after SCI. (D) Delta (Δ) values between week 1 and week 6 confirm sustained synaptic loss at epicenter. *P < 0.05, ****P < 0.0001.

To evaluate whether the lesion volume had an impact on the measured SV2A loss, the lesion area was used to extract the ^18^F-SynVesT-1 PET signal (Fig. 3A). Similar to the analysis based on anatomically defined spinal segments (Fig. 2), a significant change in SV2A at the lesion compared with the rostral C3 segment was detected at week 1 (group effect: *F*_(3,23)_ = 175.7, *P* < 0.0001; Fig. 3B). Specifically, post hoc analysis indicated that all SCI groups displayed a significant reduction in SV2A levels (*P* < 0.0001) of −37.5 ± 5.5% for 100 kDyn, −39.2 ± 3.7% for 250 kDyn, and −41.9 ± 3.5% for 400 kDyn at the lesion area compared with sham controls of −4.0% ± 3.9%. In addition, a significant difference between 100- and 400-kDyn groups was detected at the lesion (*P* = 0.049) and between 100 and 250 kDyn at C6 (*P* = 0.0064; Supplemental Fig. 3A).

Six weeks after SCI, at the chronic phase, a comparable significant change in the SV2A signal was detected (group effect: *F*_(3,22)_ = 150.5, *P* < 0.0001; Fig. 3C). Post hoc analysis confirmed that all SCI groups displayed a significant reduction in SV2A levels (*P* < 0.0001) with a group-level reduction of −37.1 ± 2.6% for 100 kDyn, −39.8 ± 6.1% for 250 kDyn, and −42.4 ± 5.9% for 400 kDyn at the lesion compared with sham controls of −4.2% ± 3.5%. Additionally, the significant difference between 100- and 400-kDyn groups also persisted (*P* = 0.019) and at C7 between 100- and 250-kDyn groups (*P* = 0.0113; Supplemental Fig. 3B).

Finally, the temporal changes did not show any significant group effect (*F*_(3,31)_ = 0.03, *P* = 0.99; Fig. 3D; Supplemental Fig. 3C), indicating that the extent of SV2A loss is established early after injury, is severity-dependent, and remains persistent over time.

### Functional Recovery Is Proportional to Contusion Severity and Remains Incomplete

**FIGURE 4.**
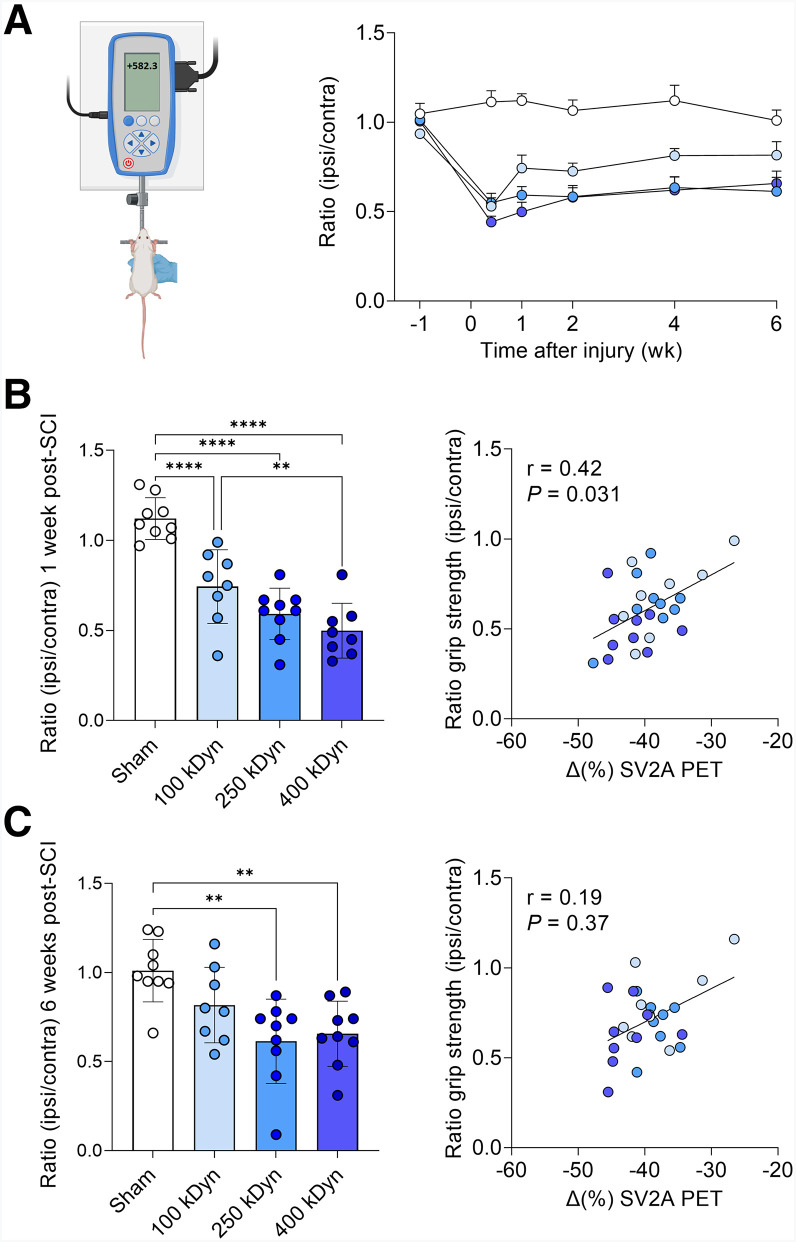
(A) Time course of ipsilateral/contralateral grip strength ratios across groups. (B) Bar plots of grip strength ratios 1 wk after injury and their correlation with 1-wk SV2A PET signal change (Δ). (C) Bar plots of grip strength ratios 6 wk after injury and their correlation with 1-wk SV2A PET signal change (Δ). Mean ± SEM. **P < 0.01, ****P < 0.0001.

To evaluate changes in functional recovery over time, grip strength was assessed throughout the postinjury period (Fig. 4A). Statistical analysis revealed a significant decrease in ipsilateral forelimb performance that depended on injury severity (interaction effect: *F*_(5,33)_ = 5.64, *P* = 0.0008; Fig. 4A). One week after injury, grip strength reduction was significant (*F*_(3,30)_ = 27.22, *P* < 0.0001; Fig. 4B), proportional to contusion severity, and correlated with in vivo PET-derived SV2A measurements (*r* = 0.42, *P* = 0.031; Fig. 4B), suggesting that early SV2A alterations might reflect the ongoing functional deficits.

At 6 wk after injury, functional recovery remained incomplete compared with controls (*F*_(3,31)_ = 7.09, *P* = 0.0009; Fig. 4C). The 250- and 400-kDyn groups exhibited comparable deficits consistent with the patterns observed in vivo with SV2A PET. No significant correlation was found between early PET measures (week 1) and functional outcome at week 6 (*r* = 0.19, *P* = 0.37; Fig. 4C), indicating that early SV2A alterations might not predict the partial functional recovery occurring over time.

### Postmortem Analysis Confirmed Severity-Dependent SV2A Loss

**FIGURE 5.**
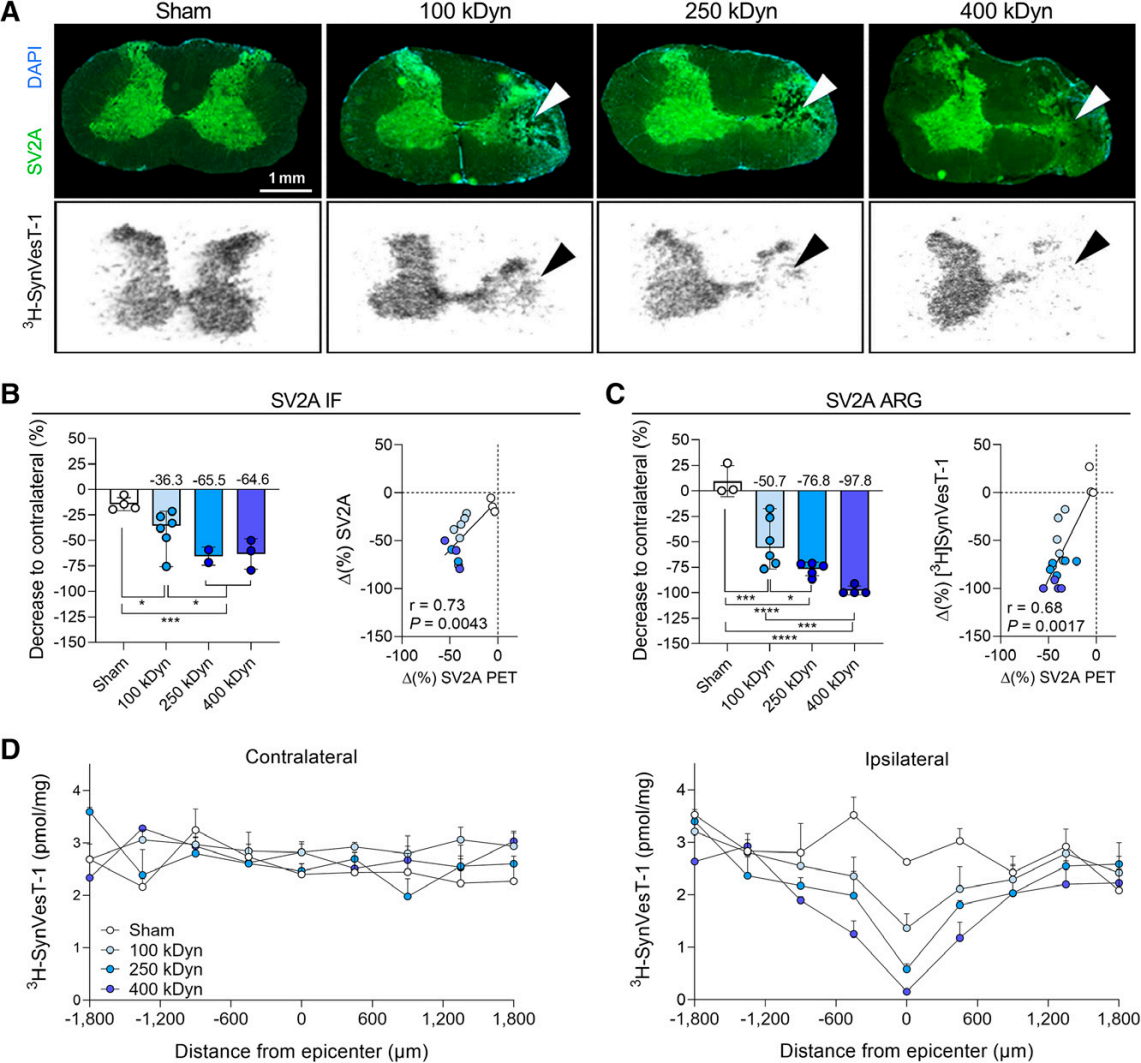
Ex vivo SV2A immunofluorescence (IF) and autoradiography (ARG) validate in vivo PET findings and confirm severity-dependent synaptic loss after SCI. (A) Representative transverse spinal cord sections showing SV2A (top row) and corresponding ^3^H-SynVesT-1 (bottom row) across injury severities. (B) SV2A immunofluorescence quantification (left) and correlation analysis with linear regression (right) of ipsilateral-to-contralateral decreases as measured by ^18^F-SynVesT-1 PET. (C) Quantification of ^3^H-SynVesT-1 and correlation analysis with linear regression of ipsilateral-to-contralateral decreases measured by ^18^F-SynVesT-1 PET. (D) Signal profiles of ^3^H-SynVesT-1 along cervical cord. *P < 0.05, ***P < 0.001, ****P < 0.0001. Δ = change; DAPI = 4′,6-diamidino-2-phenylindole.

To validate the in vivo PET findings, postmortem SV2A immunostaining and ^3^H-SynVesT-1 autoradiography analyses were performed and correlated to the in vivo data 6 wk after SCI. Representative examples depicting the severity-dependent reduction in SV2A at the epicenter are shown in Figure 5A. SV2A immunostaining revealed a significant group effect (*F*_(2,12)_ = 13.00, *P* = 0.0010; Fig. 5B) when comparing signal loss at the epicenter to the contralateral side, even though the 250- and 400-kDyn groups were pooled for statistical analysis because of the small sample size available. Nonetheless, all SCI groups displayed a substantial SV2A loss relative to the contralateral side (100 kDyn, −36.3% ± 17.7%; 250 kDyn, −65.5% ± 6.2%; 400 kDyn, −64.6 ± 12.1%) with a statistical difference between the 100-kDyn group and pooled 250/400-kDyn groups (post hoc *P* = 0.019) showing strong consistency with the in vivo SV2A PET values (*r* = 0.73, *P* = 0.0043; Fig. 5B).

Similarly, ^3^H-SynVesT-1 autoradiography showed pronounced decreases in ^3^H-SynVesT-1 binding (group effect: *F*_(3,14)_ = 28.02, *P* < 0.0001; Fig. 5C) compared with the contralateral side (100 kDyn, −51.0% ± 22%; 250 kDyn, −76.8% ± 5.9%; 400 kDyn, −97.8 ± 3.8%). Post hoc analysis further supported the observed severity-dependent SV2A loss as indicated by a statistically significant difference between the 100-kDyn groupe and both the 250-kDyn (*P* = 0.019) as well as the 400-kDyn (*P* = 0.0005) groups. Additionally, ^3^H-SynVesT-1 autoradiography correlated with the in vivo SV2A PET values (*r* = 0.68, *P* = 0.0017; Fig. 5C), and the signal profile depicted the severity-dependent SV2A loss at ipsilateral (Fig. 5D).

## DISCUSSION

This study demonstrates that ^18^F-SynVesT-1 PET imaging can detect synaptic loss after cervical contusion SCI in vivo and discriminate between low and high contusion severities. The measured SV2A reduction was not restricted to the lesion epicenter but also extended to spinal segments both rostral and caudal to the injury, suggesting detectable secondary synaptic degeneration. Notably, synaptic loss remained stable between the subacute (1 wk) and chronic (6 wk) phases, suggesting that the majority of SV2A loss occurs early and persists over time.

The pattern of synaptic loss reported is consistent with our previous work (16). Although a different SV2A PET tracer was used (^11^C-UCB-J), both studies reported similar spatial and temporal patterns in this unilateral C5 contusion SCI rat model. This suggests that synaptic loss is largely irreversible and is likely driven by excitotoxicity, neuronal loss, and microglia-mediated synaptic pruning (9). Additionally, a recent study using a thoracic contusion rat model and ^18^F-SynVesT-1 imaging supported the ability to detect synaptic loss as early as 1 d after SCI, followed by the emergence of caudal loss by day 10 (17). In our study, we observed a similar distal SV2A loss 1 wk after SCI, suggesting SV2A PET imaging is sensitive to focal and distal synaptic integrity after SCI related to primary and secondary injury. This additional delayed caudal loss may result from Wallerian degeneration, demyelination, and extension of neuronal apoptosis, with secondary mechanisms such as axonal die-back, immune cell infiltration, and gliosis further contributing to distal synaptic disruption (2,6,23). Collectively, the reliability of these findings across radioligands, injury severities, and spinal levels of contusion highlights the potential translational relevance of SV2A PET for assessing synaptic integrity in SCI.

Histologic and autoradiographic analyses confirmed SV2A presence in the spinal cord gray matter, consistent with previous observations (16). ^18^F-SynVesT-1 reliably detected synaptic loss by distinguishing between sham and all injury severities as well as between 100 and 400 kDyn. Although there was no statistically significant difference between 250 and 400 kDyn, the mean percentage decrease was greater in the 400 kDyn than in the 250 kDyn 1 wk after SCI and persisted at 6 wk after SCI. At this latter time point, ^3^H-SynVesT-1 autoradiography revealed a clear severity-dependent reductions in SV2A binding at the injury epicenter and correlated with the percentage change in SV2A PET signal. This supported the potential ability of SV2A PET to reflect severity-dependent differences between 250 and 400 kDyn, although the relatively large variability and small group differences seem to have limited the statistical power. We cannot exclude that the reduced sensitivity of in vivo PET to detect smaller effects may be limited because of its spatial resolution and potential partial-volume effects hampering its accuracy (5). This potential limitation also carries translational implications, as the small diameter of the human spinal cord gray matter might prefer the use of PET systems with extended axial length offering high spatial resolution and high sensitivity (24) to obtain an accurate evaluation of synaptic changes after injury.

Interestingly, the absence of an evident behavioral difference between the 250-kDyn and 400-kDyn groups parallels the lack of significant difference in SV2A PET signal between these severities. This suggests that the apparent plateau in both motor impairment and synaptic loss may reflect a ceiling effect, where extensive gray matter and synaptic damage beyond a certain threshold no longer translates into proportionally worse functional outcomes. These converging findings support the notion that SV2A PET provides a quantitative readout of synaptic integrity that aligns with the degree of preserved motor function. The use of only female rats in this study represents a potential limitation that may affect generalizability. Although this choice followed an established model and facilitated consistent postoperative care (20), future studies including both sexes will be important to determine whether synaptic and neurodegenerative patterns are comparable in males.

To assess volume lesion progression over time, we acquired sMRI. As expected, the volume of tissue alterations detectable with sMRI were severity-dependent at 1 wk after SCI and showed a reduction by 6 wk, likely reflecting edema clearance and progressive tissue remodeling (2). Although this was informative to confirm the severity of the injury and visualize the location of the trauma, it is reported that sMRI provides limited insight into synaptic integrity and often correlates poorly with neurologic outcomes (25–27). Alternative MRI techniques, such as diffusion tensor imaging and MR spectroscopy, can offer more detailed insights into microstructure and metabolism but are often confounded by edema and hemorrhage in the early acute phase (28). In contrast, SV2A PET directly measures synaptic density and revealed stable synaptic loss over time despite decreasing volumes of structurally affected tissue. This dissociation could suggest that chronic pathologic processes, including glial scarring and persistent inflammation, may interfere with synaptic recovery (29). Although microglia also promote circuit remodeling, these dynamic processes may not necessarily result in measurable SV2A changes, depending on the balance between synapse formation and elimination (9). The correlation between early SV2A loss with early motor impairment suggests that SV2A PET primarily detected the acute impact of synaptic disruption after SCI, whereas the weaker association of the chronic behavior with early SV2A PET pointed toward a limited predictive value for long-term recovery.

Taken together, our findings support SV2A PET as a promising noninvasive tool to monitor synaptic integrity after SCI. The stable profile of the SV2A signal suggests it primarily reflects acute, irreversible synaptic degeneration, making it a reliable baseline marker of injury severity and therapeutic response. Notably, despite the relatively mild impact of the 100-kDyn injury, we still detected significant SV2A loss, indicating that ^18^F-SynVesT-1 is sensitive to subtle synaptic changes. This sensitivity highlights the potential value of investigating even lower injury severities, such as 50 kDyn, to define the detection threshold and further validate its utility in capturing milder pathology. Additionally, SV2A PET reveals remote consequences of SCI, offering direct diagnostic and prognostic value for acute and long-term treatment guiding patient stratification.

## CONCLUSION

The present findings indicate that SV2A PET imaging with ^18^F-SynVesT-1 enables detection of SV2A loss proportional to injury severity after cervical SCI. Given the sustained severity-dependent SV2A reduction measured during subacute and chronic pathology, ^18^F-SynVesT-1 PET imaging offers a powerful noninvasive diagnostic and prognostic tool to evaluate SCI severity and monitor therapeutic effects, supporting its use in both preclinical and clinical SCI assessment.

## DISCLOSURE

This work was funded by the Wings for Life Foundation (WFL-BE-04/23) granted to Daniele Bertoglio and Charles Nicaise, and the Queen Elisabeth Medical Foundation granted to Daniele Bertoglio. Claudia Schrauwen is supported by a doctoral fellowship (1SHDG24N) from the Research Foundation Flanders (FWO). Winnok De Vos is supported by FWO (I000123N), BOF µNEURO 50208, IMPULS 50828, and AUTODIVE 52006. No other potential conflict of interest relevant to this article was reported.

## Notes

KEY POINTS **QUESTION:** Does SV2A PET imaging detect severity-dependent synaptic loss in a SCI animal model with graded severity? **PERTINENT FINDINGS:** Longitudinal ^18^F-SynVesT-1 PET imaging during subacute and chronic stages of disease demonstrated that SV2A loss is injury severity–dependent, sustained over time, and can be reliably quantified noninvasively as supported by histological and autoradiographic analyses. **IMPLICATIONS FOR PATIENT CARE:** SV2A PET imaging holds promise as noninvasive diagnostic and prognostic tool to evaluate SCI severity and monitor therapeutic interventions, supporting the translational assessment in the clinical setting for patients with SCI.

## References

[1] Alizadeh A Dyck SM Karimi-Abdolrezaee S. Traumatic spinal cord injury: an overview of pathophysiology, models and acute injury mechanisms. Front Neurol. 2019;10:282. 30967837 10.3389/fneur.2019.00282 PMC6439316

[2] Ahuja CS Wilson JR Nori S. Traumatic spinal cord injury. Nat Rev Dis Primers. 2017;3:17018. 28447605 10.1038/nrdp.2017.18

[3] Freund P Seif M Weiskopf N. MRI in traumatic spinal cord injury: from clinical assessment to neuroimaging biomarkers. Lancet Neurol. 2019;18:1123–1135. 31405713 10.1016/S1474-4422(19)30138-3

[4] Freund P Curt A Friston K Thompson A. Tracking changes following spinal cord injury: Insights from neuroimaging. Neuroscientist. 2013;19:116–128. 22730072 10.1177/1073858412449192 PMC4107798

[5] Serrano ME Kim E Petrinovic MM Turkheimer F Cash D. Imaging synaptic density: the next holy grail of neuroscience? Front Neurosci. 2022;16:796129. 35401097 10.3389/fnins.2022.796129 PMC8990757

[6] Chelyshev Y. More attention on segments remote from the primary spinal cord lesion site. Front Biosci (Landmark Ed). 2022;27:235. 36042182 10.31083/j.fbl2708235

[7] Kabdesh IM Mukhamedshina YO Arkhipova SS. Cellular and molecular gradients in the ventral horns with increasing distance from the injury site after spinal cord contusion. Front Cell Neurosci. 2022;16:817752. 35221924 10.3389/fncel.2022.817752 PMC8866731

[8] Raineteau O Schwab ME. Plasticity of motor systems after incomplete spinal cord injury. Nat Rev Neurosci. 2001;2:263–273. 11283749 10.1038/35067570

[9] Brennan FH Swarts EA Kigerl KA. Microglia promote maladaptive plasticity in autonomic circuitry after spinal cord injury in mice. Sci Transl Med. 2024;16:eadi3259. 38865485 10.1126/scitranslmed.adi3259

[10] Lane MA Fuller DD White TE Reier PJ. Respiratory neuroplasticity and cervical spinal cord injury: translational perspectives. Trends Neurosci. 2008;31:538–547. 18775573 10.1016/j.tins.2008.07.002 PMC2577878

[11] Friedli L Rosenzweig ES Barraud Q. Pronounced species divergence in corticospinal tract reorganization and functional recovery after lateralized spinal cord injury favors primates. Sci Transl Med. 2015;7:302ra134. 10.1126/scitranslmed.aac5811 PMC5669362 26311729

[12] Lynch BA Lambeng N Nocka K. The synaptic vesicle protein SV2A is the binding site for the antiepileptic drug levetiracetam. Proc Natl Acad Sci U S A. 2004;101:9861–9866. 15210974 10.1073/pnas.0308208101 PMC470764

[13] Finnema SJ Nabulsi NB Eid T. Imaging synaptic density in the living human brain. Sci Transl Med. 2016;8:348ra96. 10.1126/scitranslmed.aaf6667 27440727

[14] Bertoglio D Zajicek F De Lombaerde S. Validation, kinetic modeling, and test-retest reproducibility of [^18^F]SynVesT-1 for PET imaging of synaptic vesicle glycoprotein 2A in mice. J Cereb Blood Flow Metab. 2022;42:1867–1878. 35570828 10.1177/0271678X221101648 PMC9536120

[15] Berckmans L Schrauwen C Miranda A Staelens S Bertoglio D. Assessing non-invasive quantitative methods for [^18^F]SynVesT-1 PET imaging of synaptic vesicle glycoprotein 2A in the rat brain. Eur J Nucl Med Mol Imaging. 2025;52:3433–3443. 40032689 10.1007/s00259-025-07170-w PMC12222335

[16] Bertoglio D Halloin N De Lombaerde S. SV2A PET imaging is a non-invasive marker for the detection of spinal damages in experimental models of spinal cord injury. J Nucl Med. 2022;63:1245–1251. 35027368 10.2967/jnumed.121.263222 PMC9364338

[17] Chen B Zheng C Balayeva T. [^18^F]SynVesT-1 PET detects SV2A changes in the spinal cord and brain of rats with spinal cord injury. J Nucl Med. 2025;66:1440–1448. 40675757 10.2967/jnumed.124.269291 PMC12410297

[18] Naganawa M Li S Nabulsi N. First-in-human evaluation of ^18^F-SynVesT-1, a radioligand for PET imaging of synaptic vesicle glycoprotein 2A. J Nucl Med. 2021;62:561–567. 32859701 10.2967/jnumed.120.249144 PMC8049363

[19] Li S Naganawa M Pracitto R. Assessment of test-retest reproducibility of [^18^F]SynVesT-1, a novel radiotracer for PET imaging of synaptic vesicle glycoprotein 2A. Eur J Nucl Med Mol Imaging. 2021;48:1327–1338. 33416954 10.1007/s00259-020-05149-3

[20] Nicaise C Frank DM Hala TJ. Early phrenic motor neuron loss and transient respiratory abnormalities after unilateral cervical spinal cord contusion. J Neurotrauma. 2013;30:1092–1099. 23534670 10.1089/neu.2012.2728 PMC3689927

[21] Miranda A Bertoglio D Glorie D Stroobants S Staelens S Verhaeghe J. Validation of a spatially variant resolution model for small animal brain PET studies. Biomed Phys Eng Express. 2020;6:045001. 33444262 10.1088/2057-1976/ab8c13

[22] Everix L Zajicek F Eetveldt AV. Assessment of changes in synaptic density in the zQ175DN mouse model of Huntington’s disease: a [^18^F]SynVesT-1 study. Neuroimage Clin. 2025;46:103800. 40381377 10.1016/j.nicl.2025.103800 PMC12143836

[23] Tian R Zhou Y Ren Y Zhang Y Tang W. Wallerian degeneration: from mechanism to disease to imaging. Heliyon. 2025;11:e40729. 39811315 10.1016/j.heliyon.2024.e40729 PMC11730939

[24] Omidvari N Shanina E Leung EK. Quantitative accuracy assessment of the NeuroEXPLORER for diverse imaging applications: moving beyond standard evaluations. J Nucl Med. 2025;66:150–157. 39638433 10.2967/jnumed.124.268309 PMC11705792

[25] Ellingson BM Salamon N Holly LT. Imaging techniques in spinal cord injury. World Neurosurg. 2014;82:1351–1358. 23246741 10.1016/j.wneu.2012.12.004 PMC3980138

[26] Sharif S Jazaib Ali MY. Outcome prediction in spinal cord injury: myth or reality. World Neurosurg. 2020;140:574–590. 32437998 10.1016/j.wneu.2020.05.043

[27] Khade AB. Comparison of neurological versus functional recovery observed during rehabilitation, among paraplegics following traumatic spinal cord injury. Int J Sci Res. 2019;8:12–15.

[28] Talbott JF Nout-Lomas YS Wendland MF. Diffusion-weighted magnetic resonance imaging characterization of white matter injury produced by axon-sparing demyelination and severe contusion spinal cord injury in rats. J Neurotrauma. 2016;33:929–942. 26483094 10.1089/neu.2015.4102 PMC4876499

[29] Bradbury EJ Burnside ER. Moving beyond the glial scar for spinal cord repair. Nat Commun. 2019;10:3879. 31462640 10.1038/s41467-019-11707-7 PMC6713740

